# Quantification of Bile Acids in Cerebrospinal Fluid: Results of an Observational Trial

**DOI:** 10.3390/biomedicines11112947

**Published:** 2023-11-01

**Authors:** Lars-Olav Harnisch, Sophie Neugebauer, Diana Mihaylov, Abass Eidizadeh, Bozena Zechmeister, Ilko Maier, Onnen Moerer

**Affiliations:** 1 Department of Anaesthesiology, University Medical Center, University of Göttingen, Robert-Koch-Str. 40, D-37075 Göttingen, Germany; omoerer@med.uni-goettingen.de; 2 Institute of Clinical Chemistry and Laboratory Diagnostics, University Hospital Jena, Am Klinikum 1, D-07747 Jena, Germany; sophie.neugebauer@med.uni-jena.de (S.N.); diana.mihaylov@amedes-group.de (D.M.); 3 Interdisciplinary UMG Laboratories, University Medical Center, University of Göttingen, Robert-Koch-Str. 40, D-37075 Göttingen, Germany; abass.eidizadeh@med.uni-goettingen.de (A.E.); bozena.zechmeister@med.uni-goettingen.de (B.Z.); 4 Department of Neurology, University Medical Center, University of Göttingen, Robert-Koch-Str. 40, D-37075 Göttingen, Germany; ilko.maier@med.uni-goettingen.de

**Keywords:** bile acids, blood–brain barrier, adaptive response, active transport

## Abstract

(1) Background: Bile acids, known as aids in intestinal fat digestion and as messenger molecules in serum, can be detected in cerebrospinal fluid (CSF), although the blood–brain barrier is generally an insurmountable obstacle for bile acids. The exact mechanisms of the occurrence, as well as possible functions of bile acids in the central nervous system, are not precisely understood. (2) Methods: We conducted a single-center observational trial. The concentrations of 15 individual bile acids were determined using an in-house LC-MS/MS method in 54 patients with various acute and severe disorders of the central nervous system. We analyzed CSF from ventricular drainage taken within 24 h after placement, and blood samples were drawn at the same time for the presence and quantifiability of 15 individual bile acids. (3) Results: At a median time of 19.75 h after a cerebral insult, the concentration of bile acids in the CSF was minute and almost negligible. The CSF concentrations of total bile acids (TBAs) were significantly lower compared to the serum concentrations (serum 0.37 µmol/L [0.24, 0.89] vs. 0.14 µmol/L [0.05, 0.43]; *p* = 0.033). The ratio of serum-to-CSF bile acid levels calculated from the respective total concentrations were 3.10 [0.94, 14.64] for total bile acids, 3.05 for taurocholic acid, 14.30 [1.11, 27.13] for glycocholic acid, 0.0 for chenodeoxycholic acid, 2.19 for taurochenodeoxycholic acid, 1.91 [0.68, 8.64] for glycochenodeoxycholic acid and 0.77 [0.0, 13.79] for deoxycholic acid; other bile acids were not detected in the CSF. The ratio of CSF-to-serum S100 concentration was 0.01 [0.0, 0.02]. Serum total and conjugated (but not unconjugated) bilirubin levels and serum TBA levels were significantly correlated (total bilirubin *p* = 0.031 [0.023, 0.579]; conjugated bilirubin *p* = 0.001 [0.193, 0.683]; unconjugated *p* = 0.387 [−0.181, 0.426]). No correlations were found between bile acid concentrations and age, delirium, intraventricular blood volume, or outcome measured on a modified Rankin scale. (4) Conclusions: The determination of individual bile acids is feasible using the current LC-MS/MS method. The results suggest an intact blood–brain barrier in the patients studied. However, bile acids were detected in the CSF, which could have been achieved by active transport across the blood–brain barrier.

## 1. Introduction

Bile acids are amphiphilic molecules that are produced primarily by the liver and secreted into the intestine to aid in fat emulsification and absorption. However, not only are bile acids present in the gut but they can also be found in serum. There is increasing evidence that bile acids perform many more functions in the body than simply supporting fat emulsification in the intestine [1]. Bile acids are involved in and influence many metabolic processes and disease states [2]. Serum bile acids can be further divided into individual primary and secondary bile acids and their respective taurine and glycine conjugation products. This bile acid pattern may be even more informative than the total concentration of bile acids [3]. Bile acid receptors are present in most tissues of the body [4], where bile acids act as messenger molecules and trigger effects via membrane-bound and cytoplasmatic receptors (mainly Takeda G protein-coupled receptor 5 (TGR5)/G protein-coupled bile acid receptor-1b [GPBAR 1], Farnesoid-X receptor [FXR], and Pregnane-X receptor [PGX], among others not as specialized to bile acids). 

In addition to the extensive occupation of bile acid receptors by most organs of the body and their influence on metabolism, bile acid receptors have also been detected in the central nervous system (CNS) [5]. The reason for the presence of bile acid receptors in the CNS is unclear. Similarly, bile acids could also be detected in cerebrospinal fluid (CSF). This is particularly surprising as an intact blood–brain barrier is normally an insurmountable obstacle for bile acids [6,7,8]. However, all conceivable mechanisms for the presence of bile acids in the CNS have been proposed [6]: bile acids have been described to diffuse passively across the blood–brain barrier, with serum and brain levels correlating in a dose-dependent manner [7,8]; to be actively transported across the blood–brain barrier [9,10]; and, perhaps most surprisingly, bile acids are also produced within the CNS [11,12]. 

In many, mainly chronic, neurologic disorders, bile acid metabolism has been found to be altered, and bile acids can be detected in quantifiable and even rather high concentrations in cerebrospinal fluid and/or brain parenchyma [13,14,15,16,17]. Furthermore, even therapeutic/anti-inflammatory effects of bile acids have been described [16,17,18,19,20,21,22]. However, most of the above findings are based on experimental animal data, which limits transferability to humans due to the different spectrum of bile acids in rodents (e.g., the presence of muricolic, murideoxycholic, and hyodeoxycholic acid, which do not exist in the human organism). Therefore, the insights from these studies are not without limitations and uncertainty regarding the explanations for the occurrence of bile acids in the CNS and the pathophysiological and clinical effects. As of now, no data on the concentration of bile acids, let alone the bile acid profile of the human CSF, are available.

Recently, we were able to show that in a cohort of patients with acute respiratory distress syndrome (ARDS), the profile of individual bile acids in serum can provide valuable information on pathophysiological conditions and their development compared to the total amount of bile acids or one individual bile acid alone [3]. 

With our previous work, the view that an increase in serum bile acids in critical illness is akin to collateral damage was challenged. Therefore, the rationale for studying the present cohort was twofold: On the one hand, we want to verify our previously published findings in another critically ill cohort. On the other hand, and against the background of the above-mentioned presence of receptors for bile acids in the CNS, we are interested in whether—and if so in which concentration—bile acids are detectable in the CSF, and after determining the profile of individual bile acids with the currently available LC-MS/MS method, whether it is even possible to draw conclusions on the mechanisms of crossover in a cohort of acute and/or severe cerebral pathology.

## 2. Materials and Methods

We collected specimens from 54 patients who underwent external ventricular drainage (EVD) for various clinical reasons. Specimens were taken within 24 h after EVD placement, and blood samples were taken simultaneously.

The analysis of individual bile acid concentrations was performed at the Institute of Clinical Chemistry and Laboratory Diagnostics of the Jena University Hospital, and the determination of S100 concentrations was performed in the central laboratory of the University Medical Center Göttingen. 

Bile acid standards for each individual bile acid metabolite were purchased from VWR International GmbH (Darmstadt, Germany), TCI Deutschland GmbH (Eschborn, Germany) and Sigma-Aldrich Chemie GmbH (Taufkirchen, Germany) and were at least of 91% purity. HPLC-grade methanol, ethanol, ammonium acetate, and formic acid were obtained from Carl Roth (Karlsruhe, Germany), Merck KGaA (Darmstadt, Germany) and Sigma-Aldrich Chemie GmbH (Taufkirchen, Germany). Calibration standards were produced with a bile acid-free matrix (serum or CSF) with a concentration range of 0.05–50 µM.

Blood and CSF samples were analyzed for the concentration of primary bile acids and their metabolites: cholic acid (CA), taurocholic acid (TCA), glycocholic acid (GCA), chenodeoxycholic acid (CDCA), taurochenodeoxycholic acid (TCDCA), and glycochenodeoxycholic acid (GCDCA), and secondary bile acids and metabolites: deoxycholic acid (DCA), taurodeoxycholic acid (TDCA), glycodeoxycholic acid (GDCA), ursodeoxycholic acid (UDCA), tauroursodeoxycholic acid (TUDCA), glycoursodeoxycholic acid (GUDCA), litocholic acid (LCA), taurolitocholic acid (TLCA), and glycolitocholic acid (GLCA). CSF samples were drawn under sterile precautions from the drainage system into an unspecified sample vessel and immediately centrifuged (4500 rpm, 10 min, 20 °C). Plasma samples (silicate coagulation activator) were centrifuged 30 min after drawing the sample (4500 rpm, 10 min, 20 °C). Then, 200 µL of serum/CSF was then stored at −80 °C until further handling. Final analysis of individual bile acids in serum samples precipitated by protein and CSF was performed using an in-house LC-MS/MS method at Jena University Hospital (Institute of Clinical Chemistry and Laboratory Diagnostics). Details of the method have previously been described [3]. In summary, sample preparation was carried out by precipitating proteins with 85% aqueous methanol and filtering samples in a Thomson Single Step^®^ Filter Vial (PES membrane 0.2 µM, Thomson Instrument Company, Oceanside, CA, USA). For quantification, an Agilent 1200 high-performance liquid chromatography system (Agilent Technologies GmbH, Waldbronn, Germany) with a CTC-PAL autosampler coupled to an API 4000 Triple Quadrupole mass spectrometer with an electrospray ionization source (AB Sciex, Darmstadt, Germany) was used. All chromatographic separations were performed with a reverse-phase Agilent Zorbax Eclipse XDB-C18 (3.5 µm, 100 × 3 mm) analytical column equipped with a guard column (C18, 4 × 3 mm; Phenomenex, Aschaffenburg, Germany). The mobile phase consisted of water (A) and methanol (B), both containing 0.012% formic acid and 5 mM ammonium acetate, at a total flow rate of 300 µL/min.

Furthermore, S100 was quantified from the same blood and CSF samples using the LIAISON^®^ chemiluminescent immunoassay and analyzer system (DiaSorin S.p.A., Saluggia, Italy). The following parameters were taken from the department’s patient-data management system: age, sex, clinical diagnosis/reason for EVD, duration of EVD implantation to sample drawn, mortality, delirium, and modified Rankin Scale, as well as the biomarkers bilirubin (conjugated and unconjugated), Aspartate-Aminotransferase (AST), Alanine-Aminotransferase (ALT), Gamma-Glutamyltransferase (GGT), alkaline phosphatase (AP), albumin, albumin quotient and red blood cell count (RBC) in CSF. 

In addition, intraventricular blood volume was estimated from computed tomography scans.

Statistical analysis was performed using SPSS (International Business Machines Corporation (IBM), Armonk, NY, USA, Version 28.0). Data were tested for normal distribution using the Shapiro–Wilk test and presented as mean values with a standard deviation or median with a 95% confidence interval (CI); where appropriate, data were winsorized to account for outliers [23]. Differences between serum and CSF concentrations were tested using the Wilcoxon test, correlations were tested using the Spearman test, and causation was tested using linear regression. Statistical significance was assumed at *p* = 0.05. Informed consent was obtained from all patients and next of kin, respectively. This observational study was approved by the Institutional Review Board of Georg-August University Göttingen on 6 February 2018 (IRB No.: 24/2/18).

## 3. Results

Specimens of 54 patients who received external ventricular drainage for clinical reasons (19 intracerebral hemorrhage (of which 16 had intraventricular hemorrhage), 17 subarachnoid hemorrhage, 9 traumatic brain injury (4 isolated), 3 cerebral ischemia, 3 acute hydrocephalus, 2 elective perioperatively, 1 meningitis) were analyzed. Of these patients, 34 had undergone at least one neurosurgical procedure before the CSF sample was taken (11 ICP sensor implantation, 6 decompressive hemicraniectomy, 6 catheters for thrombolysis, 6 aneurysm clippings, 4 aneurysm coilings, 4 elective neurosurgical procedures). None of the included patients had impaired liver function or cholestasis according to the patient history and baseline laboratory values; for patient characteristics and baseline laboratory values, see Table 1. 

Serum bile acid concentrations were very low; we found almost exclusively primary bile acids. Of the secondary bile acids, only deoxycholic acid was detected, and this was also mainly in its glycine-conjugated form; the glycine-to-taurine conjugation ratio (G/T-ratio) for TBA was 3.30 [1.79, 4.79] (Table 2). The concentration of bile acids in the CSF was minute; bile acids were detected only in the CSF of 11 of the 54 investigated patients (20.4%) (Table 2).

**Figure 1:**
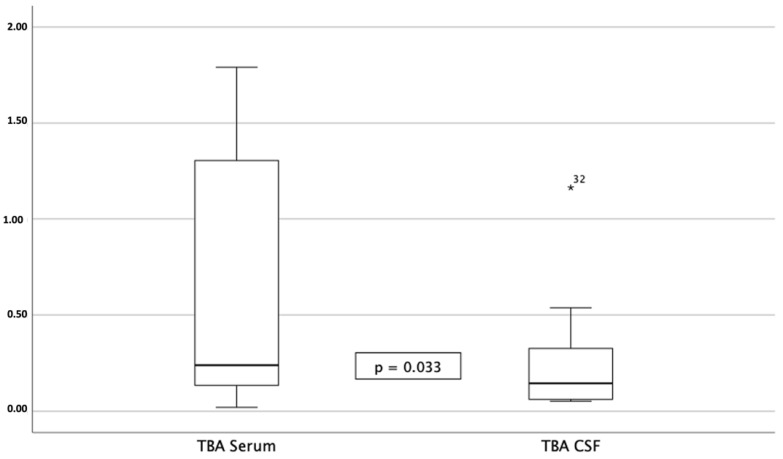
Total bile acid concentrations in serum vs. cerebrospinal fluid; the difference is statistically significant; no statistically significant correlation between concentrations in these two compartments was detected.

Total bile acid CSF concentrations differed significantly from serum TBA concentration (serum 0.37 µmol/L [0.24, 0.89] vs 0.14 µmol/L [0.05, 0.43]; *p* = 0.033) (Figure 1); bile acid concentrations in serum and CSF were not correlated. However, the total concentration of CSF bile acids was correlated with serum GGT (*p* = 0.041 [0.013, 0.876), r = 0.595) and serum AP (0.007 [0.225, 0.923), r = 0.732). The ratio of serum-to-CSF bile acid levels calculated from the respective total concentrations was 3.10 [0.94, 14.64] for TBA, 3.05 for TCA, 14.30 [1.11, 27.13] for GCA, 0.0 for CDCA, 1.91 [0.68, 8.64] for GCDCA and 0.77 [0.0, 13.79] for DCA; for all other bile acids, no ratio was calculable. Bile acid concentrations in the CSF were not correlated with the appearance of blood–brain barrier dysfunction, assessed by the albumin quotient. The ratio of CSF-to-serum S100 concentrations was 0.01 [0.00, 0.02]; likewise, the physiological ratio of CSF-to-serum concentration was maintained. These results suggest an intact blood–brain barrier, arguing against passive overflow. 

Serum total and conjugated (but not unconjugated) bilirubin levels and serum TBA levels were significantly correlated (total bilirubin *p* = 0.031 [0.023, 0.579]; conjugated bilirubin *p* = 0.001 [0.193, 0.683]; unconjugated *p* = 0.387 [−0.181, 0.426]). The total amount of bile acids in both serum and CSF was strongly correlated with established serum markers of cholestasis such as GGT (serum *p* = 0.032 [0.024, 0.580], r = 0.372; CSF *p* = 0.041 [0.013, 0.876], r = 0.595) and alkaline phosphatase (AP) (serum *p* = 0.019 [0.052, 0.598], r = 0.355; CSF *p* = 0.007 [0.255, 0.823], r = 0.732). Linear regression revealed an association between GGT and AP and the total bile acid concentration in serum (GGT and TBA serum *p* = 0.08 [0.003, 0.017], r^2^ = 0.137; AP and TBA serum *p* < 0.001 [0.020, 0.040], r^2^ = 0.477). 

**Figure 2:**
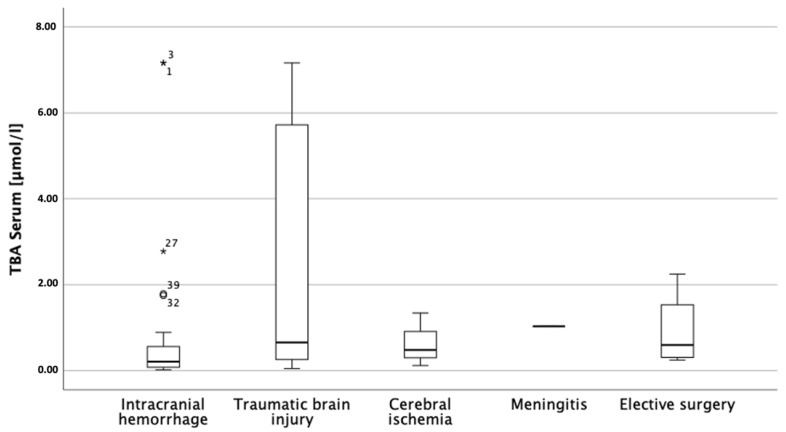
Serum total bile acid concentrations applied according to the underlying clinical picture. Although they appear to be very distinct, there were no statistically significant differences between the types of insult. This is most likely due to the relatively small number of patients.

**Figure 3:**
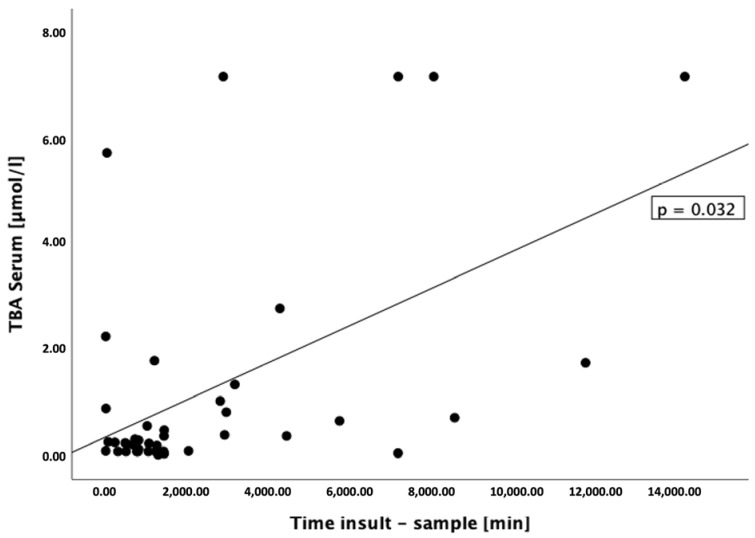
The total concentration of bile acids in serum was positively correlated with the interval of insult to sampling. Since there was no reference to cholestasis or impaired liver function, this suggests an ongoing production process.

The total concentration of serum bile acids was correlated with the type of insult (*p* = 0.032 [0.021, 0.578], r = 0.327) (Figure 2); however, the differences in the concentrations of bile acids between the groups (grouped by type of insult) were not statistically significant. No correlations were found between bile acid concentrations and sex, age, delirium, or outcome measured on a modified Rankin scale. We did not find a correlation with respect to the duration from the insertion of EVD to the time point at which the samples were drawn (*p* > 0.1), but there was a significant correlation with respect to the duration of the initial insult to the time point at which the samples were drawn and the concentration of total and glycine-conjugated bile acids in serum (TBA: *p* = 0.032 [0.020, 0.577], r = 0.327; GCA: *p* = 0.002 [0.225, 0.790], r = 0.571; GCDCA: *p* < 0.001 [0.332, 0.773], r = 0.595; GDCA: *p* = 0.036 [0.022, 0.762], r = 0.471) (Figure 3). Furthermore, significant correlations were found between the duration of the initial insult to the samples drawn and the rate of primary to secondary bile acids in serum (*p* = 0.022 [0.077, 0.807], r = 0.536) and the rate of conjugated to unconjugated serum bile acids (*p* < 0.001 [0.243, 0.710], r = 0.513), suggesting a dynamic process. Separating this further, the conjugation ratios of glycine-to-taurine (G/T-ratios) of individual bile acids were also significantly correlated with the interval from initial insult to sampling (CA: *p* = 0.012 [0.163, 0.881), r = 0.648; CDCA: *p* = 0.004 [0.324, 0.943], r = 0.782; DCA: *p* = 0.002 [0.332, 0.915], r = 0.741; UDCA: *p* = 0.019 [0.232, 0.988], r = 0.886). There were no statistically significant correlations between CSF bile acids and CSF RBC count (*p* = 0.765), CSF bile acids and the appearance of cerebral edema (*p* = 0.337), or CSF bile acids and intraventricular blood volume (*p* = 0.161), which argues largely against intraventricular blood contamination being the cause.

## 4. Discussion

The occurrence of bile acids in the CSF has already been described in animal experiments [7,9,18]. However, data from these experimental animal models are first and foremost hypothesis-generating and probably not inevitably transferable to humans. The absence of bile acids in the CSF in healthy humans was first described in the late 1970s [13]. However, recently, the presence of bile acids in human CSF has been confirmed mainly in chronic neurologic disorders [16,17,19,20,21,22]. Human data on the appearance of bile acids in acute disease states such as traumatic brain injury (TBI) or intracranial hemorrhage (ICH) are not available. Our data are the first to describe the concentrations of bile acids in serum and CSF at a variable time after injury, but within 24 hours after the placement of an EVD, determined by a LC-MS/MS method. We are able to show that in these previously neurologically, and with respect to liver function/cholestasis, healthy patients who mainly suffered from ICH or TBI, bile acids are detectable by the current method in CSF, although in minute concentrations.

We found no statistically relevant association between serum bile acid concentrations and CSF, arguing against passive overflow across the blood–brain barrier. Furthermore, serum bile acid concentrations were low and there was no statistically significant correlation between serum bilirubin as a marker of cholestasis and the bile acid concentration in the CSF, both of which also argue against passive overflow due to a strong concentration gradient, as has been described in severe cholestasis [24,25]. Furthermore, the passive transfer mechanism of bile acids above the blood–brain barrier appears to be especially true for unconjugated bile acids such as CA, CDCA, and DCA [26], which were not detected in the CSF of our cohort at all (CA) or in almost negligible minute concentrations (CDCA and DCA, respectively).

On the contrary, the high serum-to-CSF bile acid ratios we found argue in favor of an intact blood–brain barrier that repels bile acids effectively. This is reinforced by the CSF–to–serum albumin quotient, a well-established biomarker of blood–brain barrier dysfunction, which was only positive for blood–brain barrier dysfunction in 6 of the 54 patients studied and the serum-to-CSF ratio of S100, a biomarker of brain injury and blood–brain barrier dysfunction [27], which was 0.01. Furthermore, in our cohort, there was a physiological relationship between CSF and serum concentrations of S100 [28]. Therefore, the evaluation of this parameter in the aforementioned manner suggests an almost complete prevention of transfer of S100 from CSF to serum, which is also a strong hint toward an intact blood–brain barrier and against the passive overflow of bile acids and contamination by intraventricular blood, respectively.

These results might appear contrary to what has been known for a while, namely that TBI and cerebral ischemia, as well as other diseases, temporarily disrupt the blood–brain barrier [29,30]. However, a thorough examination of the literature on this issue revealed that the evidence is not as clear as initially supposed. Furthermore, the mechanisms and timing of blood–brain barrier dysfunction are neither clear nor consistent among studies [30]. Additionally, much of the research on which we base our knowledge of BBB dysfunction has been conducted in experimental animal trials and various experimental models, severely limiting the transferability [30]. Especially since many of these trials have been conducted in rodents, which have vastly different brain sizes, much more important is that rats and mice are lissencephalic, whereas humans are obviously gyrencephalic. Regarding the timing of BBB dysfunction following an insult, results range from only minutes [31] to years [32]; some researchers also describe multiphasic dysfunctions [29]. In addition, many trials rely on surrogates to assess BBB dysfunction, all of which have drawbacks. So, considering how we interpret the literature, we cannot be sure beyond doubt, how, when, and to what degree blood–brain barrier dysfunction occurs in these diseases. 

Bile acid concentrations did not differ between patients with and without intraventricular blood and there were no statistically significant correlations between bile acids in CSF and intraventricular blood volume and RBC count, respectively. This is also reinforced by the fact that no significant correlation was found between bile acid concentrations in serum and CSF, as previously stated.

Our results make passive diffusion, which also includes the contamination of blood, of serum bile acids into the CSF unlikely due to the factors mentioned. However, since bile acids in the CSF were found to increase, they need to be actively transported above the blood–brain barrier or produced within the brain parenchyma. The production of the bile acids that we found (as well as any bile acid) within brain tissue is very unlikely; because of the enzymes available for the production of bile acids in the brain, it can only be called a fragment of a synthesis pathway, which is probably only good for facilitating cholesterol turnover in the brain [14,33].

The exclusion of these two options—passive diffusion and production of bile acids in the brain—leaves us with the third option: the active transport of bile acids from the serum across the blood–brain barrier into the brain. In rats, receptors of the OATP family have been found in epithelial and endothelial cells of the brain capillaries and the choroid plexus. Receptors in this family are capable of transporting conjugated and unconjugated bile acids [34,35,36,37]; no human data are available on this topic. To date, it is also not known how these receptors are regulated in terms of the quantity and selectivity of transported bile acids, but interactions with Pregnane-X and Farnesoid-X receptors, both also activated by bile acids, have been described [5,38]. These receptors have been shown to actively influence the blood–brain barrier in the sense of lower permeability [39,40] and increase central sympathetic outflow, apparently with the aim of increasing metabolism [5]. Both of these mechanisms could be part of an adaptive reaction triggered by bile acids acting as messengers. For example, on the one hand, if a serious infection is considered, the brain might use these mechanisms to protect itself from the passage of toxins across the blood–brain barrier (PXR effect), and on the other hand, it could use them to boost metabolism to fight the infection (FXR effect). This adaptive reaction is likely not specific to the type of critical illness but more general in nature in the sense of a uniform systemic response pattern to severe illness (SIRS-like [systemic inflammatory response syndrome]). 

The correlation between the total concentration of bile acids in serum and GGT, as well as the alkaline phosphatase (AP) that we found, could also be interpreted in this sense. Since all of these biomarkers (bilirubin, GGT, AP) were within their respective reference intervals, as were AST and ALT, hepatitis, relevant liver injury, and significant cholestasis can be excluded by definition [41,42]. Another explanation would be a mechanism in which these substances, which are usually used as biomarkers to detect pathological states, are actively up-regulated. For example, AP has recently been found to have pronounced anti-inflammatory effects by inhibiting bacteria by dephosphorylating lipopolysaccharide and flagellin [43,44,45]. Furthermore, serum GGT concentration has been correlated with total serum bile acid concentration in the early phase of ARDS, a serious critical disease with high mortality [46]. ARDS is a critical condition that in turn is caused by another serious condition, such as massive injury or severe infection/sepsis. Although our group reported that bile acids have already increased on the day of ARDS diagnosis, this diagnosis often takes time to develop. The conditions we investigated in this study are much more acute in terms of the time between the triggering event and the critical condition. We took the sample at a median time of 19.75 hours after the triggering event. Therefore, a possible adaptive response with respect to the up-regulation of bile acid metabolism could not have fully taken effect for serum bile acids to be measured as elevated. This is supported by the fact that we found a significant positive correlation between insult time and the sample taken with respect to the concentration of total serum bile acids.

The clinical implications of our findings include that GGT and AP are easily available and relatively cheap screening tools for serum bile acids. Serum bile acids, in turn, may provide clues to the time course of cerebral disease, e.g., the timing of SAB in the absence of a history or the presence of an incomplete history. 

Regarding future directions in research in this field to elucidate this hypothesis, studies involving serum and, ideally, CSF samples as close to a critical event as possible, as well as serial samples over the course of the critical condition, are needed. Furthermore, because we propose a uniform systemic response, which is mediated by bile acids, different critical conditions with appropriately powered patient numbers should be investigated. Finally, research is needed on the expression of bile acid transporters in cerebral blood vessels, as well as the receptor occupancy of different cells in the brain, their respective activation mechanisms, and the molecular mechanisms of the effects they trigger in humans. Therefore, this investigation must be seen as a first hint of a possible explanation for the occurrence of bile acids in CSF and is a proof-of-principle hypothesis-generating study, and there is more research on the way. 

## 5. Conclusions

The determination of 15 individual bile acids is feasible using the current LC-MS/MS method; bile acids could be detected in the CSF of 1/5 of the patients investigated. 

The CSF-to-serum ratio of bile acids, as well as the biomarkers of blood–brain barrier dysfunction investigated, suggest an intact blood–brain barrier in the patients studied. Therefore, active transport across the blood–brain barrier would be an obvious hypothesis for this finding, which needs confirmation in further trials.

## Figures and Tables

**Table 1:** Patient characteristics.

Parameter	Value
Age (years)	60 [54, 64]
Female:male	23:31
Delirium no. (%)	11 (20.37)
Time insult–sample (h)	19.75 [9.25, 47.0)
Time EVD–sample (min)	630 [300, 810]
Bilirubin (mg/dL)	0.6 [0.5, 0.8]
Bilirubin conjugated (mg/dL)	0.3 [0.3, 0.5]
Bilirubin unconjugated (mg/dL)	0.3 [0.3, 0.4]
Aspartate-Aminotransferase (U/L)	25 [23, 31]
Alanine-Aminotransferase (U/L)	17 [15, 27]
Gamma-Glutamyltransferase (U/L)	30 [21, 50]
Alkaline phosphatase (U/L)	68.5 [61, 80]
Albumin serum (mg/dL)	2.95 [2.70, 3.20]
Red blood cells (RBCs) in CSF (n)	3521 [1514, 5322]
Intraventricular blood volume (mL)	313.5 [149, 2176]
Cerebral edema in CT no. (%)	21 (39%)

**Table 2:** Results of determination of individual bile acids and conjugation products, respectively. Abbreviations: CI = confidence interval, CSF = cerebrospinal fluid, TBA = total bile acids, CA = cholic acid, TCA = taurocholic acid, GCA = glycocholic acid, CDCA = chenodeoxycholic acid, TCDCA = taurochenodeoxycholic acid, GCDCA = glycochenodexycholic acid, DCA = deoxycholic acid, TDCA = taurodeoxycholic acid, GDCA = glycodeoxycholic acid, UDCA = ursodeoxycholic acid, TUDCA = tauroursodeoxycholic acid, GUDCA = glycoursodeoxycholic acid, LCA = litocholic acid, TLCA = taurolitocholic acid, GLCA = glycolitocholic acid, n.d. = not detected, n/a = not available.

**Bile Acid Serum (µmol/L)**	**Median [95% CI]**	**Bile Acid CSF (µmol/L)**	**Median [95% CI]**
TBA serum (µmol/L)	0.37 [0.24, 0.89]	TBA CSF (µmol/L)	0.14 [0.05, 0.43]
CA serum (µmol/L)	0.09 [0.07, 1.01]	CA CSF (µmol/L)	n.d.
TCA serum (µmol/L)	0.21 [0.08, 0.33]	TCA CSF (µmol/L)	n.d.
GCA serum (µmol/L)	0.20 [0.15, 0.39]	GCA CSF (µmol/L)	0.06
CDCA serum (µmol/L)	0.12 [0.08, 0.16]	CDCA CSF (µmol/L)	0.08 [0.07, 0.08]
TCDCA serum (µmol/L)	0.20 [0.13, 0.79]	TCDCA CSF (µmol/L)	0.07 [0.05, 0.09]
GCDCA serum (µmol/L)	0.21 [0.14, 0.54]	GCDCA CSF (µmol/L)	0.36
DCA serum (µmol/L)	0.14 [0.08, 0.23]	DCA CSF (µmol/L)	0.07 [0.06, 0.17]
TDCA serum (µmol/L)	0.16 [0.14, 0.48]	TDCA CSF (µmol/L)	0.06 [0.05, 0.09]
GDCA serum (µmol/L)	0.17 [0.12, 0.31]	GDCA CSF (µmol/L)	0.09
UDCA serum (µmol/L)	0.05	UDCA CSF (µmol/L)	0.08 [0.08, 0.23]
TUDCA serum (µmol/L)	n.d.	TUDCA CSF (µmol/L)	n.d.
GUDCA serum (µmol/L)	0.16 [0.10, 0.28]	GUDCA CSF (µmol/L)	n.d.
LCA serum (µmol/L)	n.d.	LCA CSF (µmol/L)	n.d.
TLCA serum (µmol/L)	n.d.	TLCA CSF (µmol/L)	n.d.
GLCA serum (µmol/L)	n.d.	GLCA CSF (µmol/L)	n.d.
G/T-ratio TBA serum	3.30 [1.79, 4.79]	G/T-ratio TBA CSF	n/a
Ratio Serum/CSF TBA	3.10 [0.94, 14.64]		
Ratio Serum/CSF CA	n/a		
Ratio Serum/CSF TCA	3.05		
Ratio Serum/CSF GCA	14.30 [1.11, 27.13]		
Ratio Serum/CSF CDCA	0.0		
Ratio Serum/CSF TCDCA	2.19		
Ratio Serum/CSF GCDCA	1.91 [0.68, 8.64]		
Ratio Serum/CSF DCA	0.77 [0.0, 13.79]		
Ratio Serum/CSF TDCA	n/a		
Ratio Serum/CSF GDCA	n/a		
Ratio Serum/CSF UDCA	n/a		
Ratio Serum/CSF TUDCA	n/a		
Ratio Serum/CSF GUDCA	n/a		
Ratio Serum/CSF LCA	n/a		
Ratio Serum/CSF TLCA	n/a		
Ratio Serum/CSF GLCA	n/a		

## Data Availability

The data presented in this study may be made available at reasonable request from the corresponding author.
